# Potentiating and synergistic effect of grapefruit juice on the antioxidant and anti-inflammatory activity of aripiprazole against hydrogen peroxide induced oxidative stress in mice

**DOI:** 10.1186/s12906-018-2169-x

**Published:** 2018-03-23

**Authors:** Seema Zargar, Abdul-Rahman A. Al-Majed, Tanveer A. Wani

**Affiliations:** 1 Department of Biochemistry, College of Science, King Saud University, PO Box 22452, Riyadh, 11451 Saudi Arabia; 2 Department of Pharmaceutical Chemistry, College of Pharmacy, King Saud University, Riyadh, 11451 Saudi Arabia

**Keywords:** Oxidative stress, Anti-inflammatory, Cytokines, Hydrogen peroxide, Aripiprazole, Grapefruit juice

## Abstract

**Background:**

Dependence on antipsycotic drugs like aripriprazole (ARI) is increasing at alarming rate, hence, this study was undertaken to support the hypothesis that supplementation of *Citrus paradisi* **(**Grapefruit) juice having high concentration of polyphenols might potentiate and synergize the therapeutic effect of ARI, by increasing its bioavailability and inherent antioxidant potential. These benefits together might decrease the daily dosage of the ARI and thus alleviate the possible side effects of drug.

**Methods:**

In this study the antioxidant and anti-inflammatory potential of ARI alone and in combination with GFJ was evaluated for hydrogen peroxide (H_2_O_2_) induced oxidative stress in mice. Seventy mice (4 weeks old), were randomly divided into seven groups. Group I: Control; Group II: H_2_O_2_ treated; Group III; ARI treated; Group IV GFJ treated; Group V: GFJ and H_2_O_2_ treated; Group VI; ARI and H_2_O_2_ treated; Group VII; ARI, GFJ and H_2_O_2_ treated. Serum levels of alanine aminotransferase (ALT), blood urea nitrogen (BUN), creatinine kinase (CK), creatinine and total protein were measured. Furthermore, pro-inflammatory cytokines Interleukin (IL)-1α, IL-2, IL-10 and tumor necrosis factor-α (TNF-α) concentrations were also measured.

**Results:**

The mice group that was treated with ARI, GFJ or combination of the two showed significant improvement in the H_2_O_2_ altered parameters with the combination group showing more significant improvement than the ARI and GFJ alone groups indicating a synergistic and potentiating effect of the antioxidant and anti-inflammatory potential of GFJ on ARI.

**Conclusion:**

Supplementing GFJ to ARI might increase an anti-oxidative potential of ARI due to inherent antioxidant and anti-inflammatory activity of GFJ and thus could alleviate the possible dosage dependent side effects of ARI.

## Background

Aripiprazole’s (ARI) antipsychotic activity is unique as it has an agonistic activity on presynaptic dopamine auto-receptor and an antagonistic effect on the postsynaptic dopamine D_2_ receptor [1]. This partial agonism and antagonism is due to the high affinity of ARI towards the 5-HT_1A_ and 5-HT_2A_ receptors present on serotonin neurons. ARI has been recommended for the treatment of schizophrenia and bipolar disorders. [2] Also, ARI is used for neuropsychiatric symptoms, commonly seen in Alzheimer disease, but results from randomized controlled trials on the safety and efficacy of these drugs are conflicting. In spite of that, the use of ARI is rapidly increasing throughout the world because it shows modest efficacy in the treatment of Alzheimer disease related psychosis [3]. Furthermore, ARI is more effective for particular symptoms, such as anger and aggression [4].

Phospholipids are present in very high amounts in the central nervous tissue. The phospholipids present include docosahexaenoic acid, arachidonic acid, inositol phosphate and diacylglycerol, and these can be peroxidated on exposure to reactive oxygen molecules, generating reactive oxygen species (ROS). Neurotransmitters such as dopamine, serotonin, glutamate and acetyl choline generate these phospholipids as secondary messengers that are pathological reasons for the disease of central nervous system [5].

ROS may cause damage to nucleic acids, denature proteins, and saturating the fatty acids in lipid bilayer, resulting in altered membrane structure and function [6]. Since the demand for ATP is high, the brain consumes oxygen (O_2_) rapidly, and is thus susceptible to interference with mitochondrial function, which can in turn lead to increased O_2_^−^ formation. The brain contains multiple antioxidant defenses to restrict the harmful effects of ROS, of which the mitochondrial manganese-containing superoxide dismutase and reduced glutathione seem especially important [6–9]. In depression there is loss of innate antioxidant activity mechanisms and pro-inflammatory cytokines that in turn result in the increased free radical formation because of phagocytic cell activation [10]. Hydrogen peroxide (H_2_O_2_) has been used to produce the mice models to mimic oxidative stress [11].

Many studies have reported the association between the depression and activation of anti-inflammatory responses. The inflammatory response is demonstrated by the increased production of the pro-inflammatory cytokines, such as interleukins and tumor necrosis factor-α. It has been proved in animal and human models that changes in these pro-inflammatory cytokines produces behavioral changes leading to depression. These behavioral changes include lack of good feeling, weight loss, insomnia, social withdrawal, anorexia, psychomotor retardation, fatigue and irritability. Anti-inflammatory and pain relief effects with antidepressant treatments by unknown mechanism is well documented [5, 12].

The role of ROS has been established in various neurological and psychiatric diseases as central nervous system is highly vulnerable to oxidative stress. Treatment with antidepressants may suppress immune cells which in turn could result in reduction of ROS production. ARI has been reported to have antioxidant potential [10]. Flavonoid compounds are widely distributed in plant kingdom and citrus fruits contain high content of flavonoids [13]. Flavonoids in citrus fruits and their metabolites possess antioxidant and anti-inflammatory properties, thus, reducing the risk for various chronic diseases mediated through ROS [13–15]. Grapefruit (*Citrus paradisi*; family: Rutaceae) is rich in citrus flavonoids and has beneficial anti-oxidant and anti-inflammatory properties [16–21]. Grapefruit juice (GFJ) has been shown to have antioxidant potential in mice [16, 17]. The protective effect of GFJ against genotoxicity has also been proved in various other studies [16, 18, 19]. Several studies have shown the protective effect of GFJ against hydrogen peroxide induced toxicity because of its antioxidant potential [20, 21]. High amounts of naringin flavonoid (1 mmol l^− 1^) has been found and is unique to GFJ [22]. GFJ is also known for its pharmacokinetic drug interactions as it increases the plasma concentration of several drugs by inhibiting the metabolism through inhibition of the cytochrome P450 isoforms [23, 24]. The orange juice lacks naringin and also does not interact with the pharmacokinetics of other drugs attributing most of its interaction properties to naringin [25, 26]. Naringin in GFJ has also been shown to possess antioxidant and anti-inflammatory activities. In addition to naringin a number of other flavonoids have been reported in the grapefruit juices Table 1 [27]. GFJ due to its intrinsic antioxidant and anti-inflammatory activities can potentiate and/or synergize the activity of ARI, hence the synergistic and potentiating effect of GFJ on the antioxidant and anti-inflammatory potential of ARI was studied in different H_2_O_2_ groups of mice by evaluating the biochemical and inflammatory oxidative stress parameters.

**Table 1:** Flavonoid content in grape fruit juice [27]

Flavonoid	Range (mg/100 mL)
Naringin	10.1–86.7
Narirutin	2.6–12.2
Naringenin	0–12.6
Poncirin	0.1–1.9
Hesperidin	1.6–3.1
Didymin	0.3–1.7
Neohesperidin	0.2–1.1
Quercetin	0.2–0.9

## Methods

### Chemicals

All the chemicals used including ARI and naringin, were purchased from Sigma Chemical C., St Louis, MO, USA. ELISA kits were purchased from R&D Systems, Minneapolis, MN, USA. Kits for biochemical estimations were procured from Human Diagnostic Worldwide; Wiesbaden, Germany.

### Preparation of grapefruit juice

The grapefruit used in the study was of imported to Saudi Arabia from United States origin and purchased from Tamimi Markets, Arqah, Riyadh, Saudi Arabia. GFJ was extracted by a domestic squeezer (Braun Citromatic Pulp Control MPZ6) and was filtered using a sieve of mesh diameter 1 mm.

### Animals

Healthy 4 to 5 week old mice (male) were obtained from the Animal Breeding Facility of King Saud University, Riyadh, Saudi Arabia and kept in the Central Animal House Facility of the institute. Mice were kept in polypropylene cages at room temperature with relative humidity of 60 ± 15%, and 12 h light-dark cycle. The study was approved by the Institutional Animal Ethics Committee of King Saud University.

### Experimental protocol

Seventy healthy male mice weighing between 25 and 30 g were randomly divided into seven groups (*n* = 10 each) and treated daily for 4 weeks as follows: Group I (G-I) control group (normal saline); Group II (G-II) H_2_O_2_ treated; Group III (G-III) 2 mg ARI along with normal drinking water; Group IV (G-IV) 0.5 ml of GFJ with normal drinking water; Group V (G-V) 2 mg/kg ARI with H_2_O_2_ in drinking water, Group VI (G-VI) 0.5 ml GFJ with H_2_O_2_ in drinking water, Group VII (G-VII) 2 mg/kg ARI and 0.5 ml GFJ juice along with H_2_O_2_ in drinking water. ARI was dissolved in 2 ml physiologic saline (0.9% *w*/*v*) and administered orally (gastric gavage). In all H_2_O_2_ treated groups normal drinking water was replaced with 0.4% H_2_O_2_. Samples of fresh juice were pasteurized by method of Tatum and Berry [28] and was given by oral gavage. All the groups were sacrificed after 24 h by asphyxiation with carbon dioxide.

### Flavonoid analysis

Reverse phase liquid chromatographic technique (RP-HPLC) [26] with some minor modifications was used to determine the content of naringin in the grapefruit juice. Briefly the chromatographic procedure included homogenization of GFJ and dimethylformamide in the ratio of 1:1 and centrifugation of the resulting solution at 4000 *g* for 20 min. The supernatant was filtered using as 0.2 μm filter and a sample of 20 μL was injected in the HPLC system (Waters, USA). The separation was carried out using as stainless steel 5 μm (Altima C18) column with dimensions of (250 × 4.6 mm I.D.). The mobile phase consisted of acetonitrile and 4% acetic acid in the ration of 65:35 *v*/v. the analysis was carried out at 280 nm and naringin was identified by comparison with the standard sample. The naringin was quantified using the known concentration of external standard. The standard calibration curve was plotted between different concentrations (0.5, 1.0, 2.0, 4.0, 8.0 and 1.6 mg mL^− 1^) of standard naringin against peak area. The concentration of the sample was determined from the calibration curve.

### Biochemical analysis

Biochemical parameters including ALT, BUN, serum creatinine, and CK were estimated using various kits based on colorimetric methods (Human Diagnostic Worldwide; Wiesbaden, Germany) according to the manufacture instructions. The serum samples prepared from each mice group were stored at − 80 °C for biochemical and cytokine analyses.

Briefly, for ALT estimation 200 μl of serum was added to 1 ml of working reagent and incubated at 37 °C for 1 min. The absorbance of solution was measured at 340 nm every 1 min for 3 min.

For serum creatinine estimation, 10 μl serum was added to 1 ml of working reagent and the samples incubated at 37 °C for 30 s. The absorbance of the creatinine sample was read at 492 nm immediately and after 2 min.

For BUN estimation 10 μl serum was added to 1 ml of working reagent and the samples incubated at 37 °C for 30 s. Absorbance was read immediately and 1 min later at 340 nm for estimation of serum urea level The serum BUN level was then calculated by dividing the serum urea level by 2.14, the conversion factor derived on the molecular weights of both BUN and urea.

Protein levels in serum were measured by using bovine serum albumin as standard [29]. The amount of protein was calculated from a standard curve. Protein values were expressed as g/dL. The levels of TNF-α, IL-1α, IL-2 and IL-10 were determined using an ELISA technique according to manufacturer’s instructions (R&D Systems, Minneapolis, MN, USA). Plasma samples were pretreated with specific reagents for indicated period and were measured for cytokines concentration in accordance with the manufacturer’s instructions with the detection limits of 3 pg/ml for TNF-α, 7 pg/ml for IL-1, 3 pg/ml for IL-2 and 3 pg/ml for IL9–10. The optical density of the samples was determined using a microplate reader set at 450 nm.

### Statistical analysis

The results were expressed as mean ± SE. The data was analyzed using ANOVA – Tukey post Hoc multiple comparison test. All statistical analysis was performed using Graph-Pad prism 6.0 software. *P* < 0.05 was considered statistically significant difference.

## Results

**Fig. 1:**
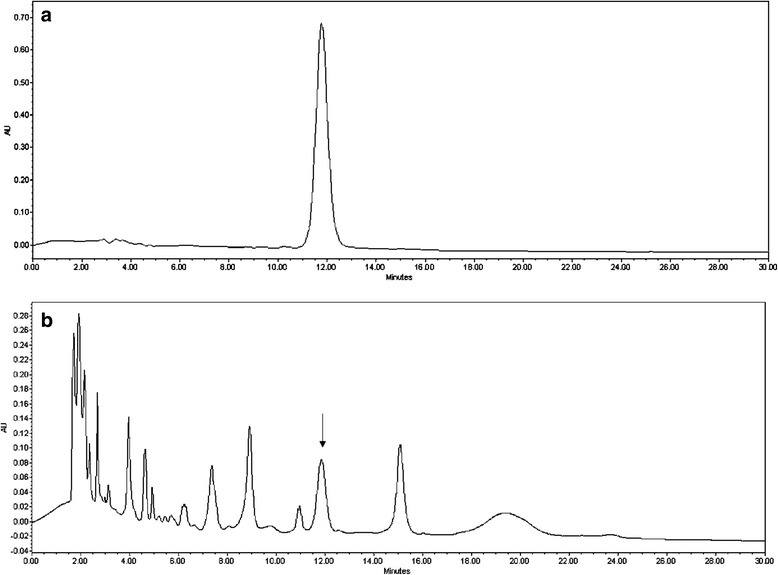
**a**; Representative chromatogram of standard drug naringin; **b**; Representative chromatogram of the 50 μL GFJ injected into HPLC system

The content of naringin, the principal flavonoid present in GFJ, responsible for its anti-inflammatory, antioxidant and drug interaction properties was estimated using RP-HPLC technique. The concentration was found to be 0.39 mg mL^− 1^ (Fig. 1).

To evaluate the protection against oxidative stress and inflammation by ARI and GFJ alone and in combination in H_2_O_2_ treated mice serum levels of certain biochemical markers (ALT, BUN, CK, creatinine and total protein) and pro-inflammatory cytokines (IL-1α, IL-2, IL- 10 and TNF-α) were measured. The levels of the obtained results for the biomarkers are presented in Table 2.

**Table 2:** Effect of H_2_O_2,_ GFJ, ARI induced alterations alone and in combination, on the levels of enzymatic and non-enzymatic antioxidant contents in serum of control and treated mice

Treatment Groups	Group I Control	Group II H_2_O_2_	Group III GF	Group IV ARI	Group V GF+ H_2_O_2_	Group VI ARI+ H_2_O_2_	Group VII ARI + GF + H_2_O_2_
ALT (U/ml)	40.07 ± 2.70	103.3 ± 3.40^*^	36.98 ± 3.30	38.03 ± 3.90	69.42 ± 4.29 ^*#^	49.98 ± 3.43^*#^	38.99 ± 3.29^#^
Protein (mg /ml)	4.45 ± 0.90	2.08 ± 0.20^*^	4.12 ± 0.19	4.57 ± 0.10	3.70 ± 0.70^*#^	4.38 ± 0.30^#^	4.98 ± 0.21^#^
BUN (mg/dl)	43.07 ± 2.69	129.6 ± 4.10^*^	41.50 ± 3.40	38.06 ± 2.80	73.86 ± 3.67 ^*#^	56.50 ± 2.85^*#^	40.31 ± 3.28^#^
Creatinine (mg/dl)	0.32 ± 0.03	0.58 ± 0.07^*^	0.385 ± 0.06	0.285 ± 0.01	0.48 ± 0.08^*#^	0.45 ± 0.04^*#^	0.41 ± 0.06^#^
CK (U/ml)	66.66 ± 5.30	52.6 ± 7.80^*^	63.12 ± 5.20	68.28 ± 3.60	69.9 ± 8.70^#^	71.9 ± 6.30^#^	60.53 ± 4.9

* Statistically significant when compared to control group (*P* < 0.0001)

^#^Statistically significant when compared to H_2_O_2_ treated group (*P* < 0.0001)

Data presented as (mean ± SE) of three independent experiments and No. of animals (n = 10)

A significant increase was observed in the ALT, BUN and creatinine levels whereas a decrease in CK and total protein was observed in the H_2_O_2_ treated group. There was no significant change in the ALT, BUN, CK, creatinine and total protein levels of the ARI and GFJ treated groups compared to the control group. In ARI+ H_2_O_2,_ GFJ+ H_2_O_2_ and GFJ + ARI + H_2_O_2_ treated groups there was a significant reversal in the ALT, BUN, CK, creatinine and total protein levels compared to H_2_O_2_ treated group. Amongst the three the GFJ + ARI + H_2_O_2_ group could significantly reverse the changes caused by H_2_O_2_ in ALT, BUN, creatinine and total protein levels and the changes obtained were insignificant compared to control group.

**Fig. 2:**
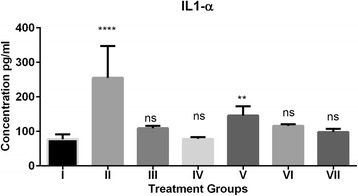
Effect of grapefruit juice (GFJ) and aripiprazole (ARI) on oxidative stress induced by H_2_O_2_ by IL-1α levels. IL-1α concentration is expressed as pg/ml (*n* = 10); The treated groups were compared to control group. ****P < 0.0001, **P < 0.01, ns: non-significant. ^#^Group I: Control; Group II: H2O2 treated; Group III; ARI treated; Group IV GFJ treated; Group V: GFJ and H_2_O_2_ treated; Group VI; ARI and H_2_O_2_ treated; Group VII; ARI, GFJ and H_2_O_2_ treated

**Fig. 3:**
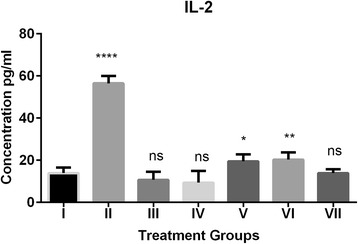
Effect of grapefruit juice (GFJ) and aripiprazole (ARI) on oxidative stress induced by H_2_O_2_ by IL-2 levels. IL-2 concentration is expressed as pg/ml (n = 10), The treated groups were compared to control group. ****P < 0.0001, **P < 0.01, *P < 0.1, ns: non-significant. ^#^Group I: Control; Group II: H2O2 treated; Group III; ARI treated; Group IV GFJ treated; Group V: GFJ and H_2_O_2_ treated; Group VI; ARI and H_2_O_2_ treated; Group VII; ARI, GFJ and H_2_O_2_ treated

**Fig. 4:**
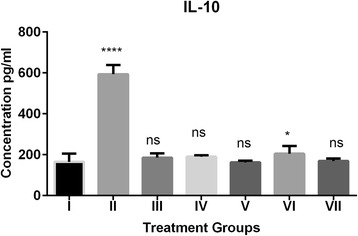
Effect of grapefruit juice (GFJ) and aripiprazole (ARI) on oxidative stress induced by H_2_O_2_ by IL-10 levels. IL-10 concentration is expressed as pg/ml (n = 10); The treated groups were compared to control group. ****P < 0.0001, *P < 0.1, ns: non-significant. ^#^Group I: Control; Group II: H2O2 treated; Group III; ARI treated; Group IV GFJ treated; Group V: GFJ and H_2_O_2_ treated; Group VI; ARI and H_2_O_2_ treated; Group VII; ARI, GFJ and H_2_O_2_ treated

**Fig. 5:**
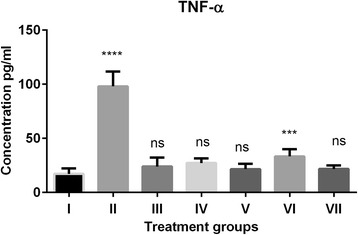
Effect of grapefruit juice (GFJ) and aripiprazole (ARI) on oxidative stress induced by H_2_O_2_ by TNF-α levels. TNF-α concentration is expressed as pg/ml (*n* = 10 The treated groups were compared to control group. ****P < 0.0001, ***P < 0.001, ns: non-significant. ^#^Group I: Control; Group II: H2O2 treated; Group III; ARI treated; Group IV GFJ treated; Group V: GFJ and H_2_O_2_ treated; Group VI; ARI and H_2_O_2_ treated; Group VII; ARI, GFJ and H_2_O_2_ treated

The pro-inflammatory factors like IL-1α, IL-2, IL-10 and TNF-α elevated due H_2_O_2_ induced oxidative stress. The elevated levels of IL-1α decreased significantly in (ARI) and (ARI + GFJ) treated groups however GFJ alone could not decrease the elevated levels of IL-1α significantly when compared to control group (Fig. 2). Similarly, the combination of ARI + GFJ decreased IL-2 significantly when compared to H_2_O_2_ group but there was insignificant difference with the control group (Fig. 3). In case of IL-10, GFJ and combination of ARI and GFJ reduced elevated levels IL-10 significantly compared to H_2_O_2_ treated group like those in control group (Fig. 4). The TNF-α levels were reversed significantly in GFJ treated group but the ARI treated group showed incomplete protection when compared to control. While as the combination of GFJ and ARI showed significant protection when compared to the control group (Fig. 5).

## Discussion

ARI, GFJ and combination of the two protects the mice from oxidative stress and inflammatory injury induced by H_2_O_2_ with reversal of altered enzyme/cytokine levels. ALT estimation is important for determination of hepatic injury and its levels were reduced significantly in mice treated with ARI and GFJ alone as well as in combination in the H_2_O_2_ treated group. The ALT levels could not reach the normal values when GFJ and ARI were used alone, however in the treatment group where the combination of GFJ and ARI was used the ALT levels decreased significantly and were insignificantly different from the control values. The possible explanation for these results could be the synergistic antioxidant property of GFJ on ARI. Since, GFJ contains citrus flavonoids and these have been known to possess diverse biologic activities. Concomitant to our study, previous studies demonstrated that elevated ALT levels decreased post antioxidant treatment [30]. Naringin and naringenin are amongst the most important citrus flavonoids and are present in abundance in GFJ and thus attribute anti-inflammatory and antioxidant properties to GFJ. [16–21, 31]. It has been hypothesized that since orange juice lacks the naringin, hence does not interact with various drugs. Therefore, the interaction with ARI with GFJ could be because of presence in high amounts of naringin in GFJ. Also, naringin has been shown to have interaction with several other drugs [32, 33].

Similarly, the total protein levels returned to normal in all the three treatment groups i.e., ARI, and ARI + GFJ group. However, GFJ alone could not increase back the total protein levels significantly compared to control group. Oxidative stress impacts translation and protein degradation, affecting protein expression levels in addition to changes at the mRNA level. The data of this study is concomitant with the previous literature which reports that translation and protein synthesis are generally down-regulated during oxidative stress depending on the type of stress [34]. Furthermore, increase or decrease in protein levels may mean that liver damage has occurred or disease is present. The BUN levels were elevated due to oxidative stress. These results are in agreement with elevated BUN levels in oxidative stress by Kojima et al... 2007 [35], who correlated the higher levels with the impairment of kidney function. ARI, GFJ and their combination significantly reduced the BUN levels. The combination of the two was more potent in bringing the BUN to normal levels compared to either of them alone. In case of CK all the treatment groups decreased the CK levels back to normal thus showing the efficacy of the three against high CK levels. Increase in CK levels in oxidative stress was also reported by Ohta et al [36].

Insult with H_2_O_2_ activates NF-kB and JNK pathways through stimulating ROS generation [37–39]. The activation of these signal pathways enhanced downstream inflammatory and oxidative reactions in liver and facilitated the production of cytokines and chemotactic factors like IL-1α, IL-2, IL-10 and TNF-α [40, 41]. IL-1 plays a crucial role in sustaining oxidative stress. The constitutive expression of IL-1 has been already shown to stimulate angiogenesis and promote tumor growth and metastasis in some mouse melanoma models [42, 43]. The elevated levels of all inflammatory cytokines reversed back significantly in the mice groups treated with ARI and GFJ.

In this study, GFJ potentiates the effect of ARI, since ARI is more potent antioxidant than GFJ as is seen in the results section. The potentiating effect of GFJ on ARI can be attributed to the fact that that GFJ increases the oral bioavailability of various drugs by inhibition of CYP3A4 and CYP2D6 iso-enzymes, thus reducing the first pass elimination of these drugs [44–46]. As ARI is metabolized by the cytochrome P450 isoforms CYP3A4 and CYP2D6 and also it’s main pharmacologically active metabolite OPC-14857 is dependent on CYP3A4 and CYP2D6 for its production and elimination [45]. Therefore, GFJ can influence the plasma concentration of ARI. Thus the GFJ could have both the synergistic antioxidant and anti-inflammatory action as well as it can also potentiate the action of ARI by increasing the plasma concentration of the ARI. The combination of GFJ and ARI was seen to be more potent effect and showed higher level of protection in all our parameters. The possible reason for greater protection from the combination could be the synergistic effect of GFJ. GFJ contains polyphenols and previous studies have reported that polyphenol compounds exert an anti-inflammatory effect on oxidative stress particularly by reducing inflammatory cytokines [47, 48]. There is very high variation in the flavonoid content of GFJ and naringin is the most abundant and unique flavonoid present in the GFJ (Table 1). Naringin has several health benefits and is bioavailable as naringin aglycone (naringenin). Naringin or naringenin acts as an antioxidant, hypolipidemic, increases alcohol metabolism, anti-apoptotic, decreases risk of atherosclerosis etc [27]. It has also been reported that pro-inflammatory cytokine production decreased post antipsychotic treatments [5]. Hence, the use of ARI along with GFJ juice might increase to the anti-oxidative potential of ARI due to inherent antioxidant and anti-inflammatory activity. GFJ could also increase the bioavailability of ARI, reducing possible side effects of ARI due to its lesser dosage.

## Conclusion

GFJ and ARI both reversed the altered biochemical and pro-inflammatory parameters in mice treated with hydrogen peroxide but none of them individually could reach to the insignificant difference with levels of control group. However, the combination of GFJ and ARI reduced all the altered parameters to insignificant differences with control group. We conclude a synergistic and potentiating effect of GFJ and ARI. Further, on the basis of results obtained, we demonstrate that the dose of ARI could potentially be reduced in combination therapy with GFJ, though further studies are needed, including trials involving humans.

## References

[1] Kikuchi T, Tottori K, Uwahodo Y (1995). A new putative antipsychotic drug with both presynaptic dopamine autoreceptor agonistic activity and postsynaptic D2 receptor antagonist activity. J Pharmacol Exp Ther.

[2] DeLeon A, Patel NC, Crismon ML (2004). Aripiprazole: a comprehensive review of its pharmacology, clinical efficacy, and tolerability. Clin Ther.

[3] De Deyn PP, Drenth AF, Kremer BP, Oude Voshaar RC, Van Dam D (2013). Aripiprazole in the treatment of Alzheimer's disease. Expert Opin Pharmacother.

[4] Schneider LS, Dagerman K, Insel PS (2006). Efficacy and adverse effects of atypical antipsychotics for dementia: meta-analysis of randomized, placebo-controlled trials. The Am J Geriatr Psychiatry.

[5] Caiaffo V, Oliveira BD, Sá FB, Evêncio Neto J. Anti-inflammatory, antiapoptotic, and antioxidant activity of fluoxetine. Pharmacol Res Perspect. 2016;4(3) 10.1002/prp2.231 PMC4876141 27433341

[6] Halliwell B (2001). Role of free radicals in the neurodegenerative diseases. Drugs Aging.

[7] Zargar S, Siddiqi NJ, Al Daihan SK, Wani TA (2015). Protective effects of quercetin on cadmium fluoride induced oxidative stress at different intervals of time in mouse liver. Acta Biochim Pol.

[8] Cutler RG. Genetic stability and oxidative stress: common mechanisms in aging and cancer. In: Free radicals and aging: Springer; 1992. p. 31–46. 10.1007/978-3-0348-7460-1_4 1450594

[9] Epstein FH, Ross R (1999). Atherosclerosis—an inflammatory disease. N Engl J Med.

[10] Eren İ, Nazıroğlu M, Demirdaş A (2007). Protective effects of lamotrigine, aripiprazole and escitalopram on depression-induced oxidative stress in rat brain. Neurochem Res.

[11] Halliwell B, Clement MV, Long LH (2000). Hydrogen peroxide in the human body. FEBS Lett.

[12] Miljević Č, Nikolić-Kokić A, Nikolić M, Niketić V, Spasić MB, Lečić-Toševski D, Blagojević D (2013). Effect of atypical antipsychotics on antioxidant enzyme activities in human erythrocytes (in vitro study). Hum Psychopharmacol.

[13] Bracke M, Bruyneel E, Vermeulen SJ, Kl V, Vanmarck V, Mareel M (1994). Citrus flavonoid effect on tumor invasion and metastasis. Food Tech.

[14] Benavente-García O, Castillo J, Marin FR, Ortuño A, Del Río JA (1997). Uses and properties of citrus flavonoids. Journal Agric Food Chem.

[15] Attaway JA (1994). Citrus juice flavonoids with anticarcinogenic and antitumor properties. ACS symposium series (USA): 1994.

[16] Alvarez-Gonzalez I, Madrigal-Bujaidar E, Martino-Roaro L, Espinosa-Aguirre J (2004). Antigenotoxic and antioxidant effect of grapefruit juice in mice treated with daunorubicin. Toxicol Lett.

[17] Ribeiro IA, Rocha J, Sepodes B, Mota-Filipe H, Ribeiro MH (2008). Effect of naringin enzymatic hydrolysis towards naringenin on the anti-inflammatory activity of both compounds. J Mol Catal B Enzym.

[18] Alvarez-Gonzalez I, Mojica R, Madrigal-Bujaidar E, Camacho-Carranza R, Escobar-García D, Espinosa-Aguirre J (2011). The antigenotoxic effects of grapefruit juice on the damage induced by benzo (a) pyrene and evaluation of its interaction with hepatic and intestinal cytochrome P450 (Cyp) 1a1. Food Chem Toxicol.

[19] Jagetia GC, Reddy TK (2002). The grapefruit flavanone naringin protects against the radiation-induced genomic instability in the mice bone marrow: a micronucleus study. Mutat Res Genet Toxicol Environ Mutagen.

[20] Razo-Aguilera G, Baez-Reyes R, Álvarez-González I, Paniagua-Pérez R, Madrigal-Bujaidar E (2011). Inhibitory effect of grapefruit juice on the genotoxicity induced by hydrogen peroxide in human lymphocytes. Food Chem Toxicol.

[21] Kanno S-i, Shouji A, Asou K, Ishikawa M (2003). Effects of naringin on hydrogen peroxide-induced cytotoxicity and apoptosis in P388 cells. J Pharmacol Sci.

[22] Kuhnau J (1976). The flavonoids: a class of semi-essential food components; their role in human nutrition. World Rev Nutr Diet.

[23] Ameer B, Weintraub RA (1997). Drug interactions with grapefruit juice. Clin Pharmacokinet.

[24] Lown KS, Bailey DG, Fontana RJ, Janardan SK, Adair CH, Fortlage LA, Brown MB, Guo W, Watkins PB (1997). Grapefruit juice increases felodipine oral availability in humans by decreasing intestinal CYP3A protein expression. J Clin Invest.

[25] Bailey DG, Spence JD, Munoz C, Arnold JMO (1991). Interaction of citrus juices with felodipine and nifedipine. Lancet.

[26] Vanamala J, Reddivari L, Yoo KS, Pike LM, Patil BS (2006). Variation in the content of bioactive flavonoids in different brands of orange and grapefruit juices. J Food Compost Anal.

[27] Zhang JI (2007). Flavonoids in grapefruit and commercial grapefruit juices: concentration, distribution, and potential health benefits. Proc Fla State Hort Soc.

[28] Tatum JH, Berry RE (1973). Method for determining naringin content in grapefruit juice. J Food Sci.

[29] Markwell MAK, Haas SM, Bieber L, Tolbert N (1978). A modification of the Lowry procedure to simplify protein determination in membrane and lipoprotein samples. Anal Biochem.

[30] Demiryilmaz I, Sener E, Cetin N, Altuner D, Suleyman B, Albayrak F, Akcay F, Suleyman H (2012). Biochemically and histopathologically comparative review of thiamine’s and thiamine pyrophosphate’s oxidative stress effects generated with methotrexate in rat liver. Med Sci Monit Basic Res.

[31] Alam MA, Subhan N, Rahman MM, Uddin SJ, Reza HM, Sarker SD (2014). Effect of citrus flavonoids, naringin and naringenin, on metabolic syndrome and their mechanisms of action. Adv Nutr.

[32] Kupferschmidt HHT, Ha HR, Ziegler WH, Meir PJ, Krahenbuhl S (1995). Interaction between grapefruit juice and midazolam in humans. Clin Pharmacol Ther.

[33] Hukkinen SK, Varhe A, Olkkola KT, Neuvonen PJ (1995). Plasma concentrations of triazolam are increased by concomitant ingestion of grapefruit juice. Clin Pharmacol Ther.

[34] Shenton D, Smirnova JB, Selley JN, Carroll K, Hubbard SJ, Pavitt GD, Ashe MP, Grant CM (2006). Global translational responses to oxidative stress impact upon multiple levels of protein synthesis. J Biol Chem.

[35] Kojima I, Tanaka T, Inagi R, Kato H, Yamashita T, Sakiyama A, Ohneda O, Takeda N, Sata M, Miyata T (2007). Protective role of hypoxia-inducible factor-2α against ischemic damage and oxidative stress in the kidney. J Am Soc Nephrol.

[36] Ohta Y, Kaida S, Chiba S, Tada M, Teruya A, Imai Y, Kawanishi M (2009). Involvement of oxidative stress in increases in the serum levels of various enzymes and components in rats with water-immersion restraint stress. J Clin Biochem Nutr.

[37] Du K, Williams CD, McGill MR, Xie Y, Farhood A, Vinken M, Jaeschke H (2013). The gap junction inhibitor 2-aminoethoxy-diphenyl-borate protects against acetaminophen hepatotoxicity by inhibiting cytochrome P450 enzymes and c-Jun N-terminal kinase activation. Toxicol Appl Pharmacol.

[38] Seki E, Brenner DA, Karin M (2012). A liver full of JNK: signaling in regulation of cell function and disease pathogenesis, and clinical approaches. Gastroenterology.

[39] Fouad AA, Jresat I (2012). Hepatoprotective effect of coenzyme Q10 in rats with acetaminophen toxicity. Environ Toxicol Pharmacol.

[40] Zimmermann HW, Trautwein C, Tacke F (2012). Functional role of monocytes and macrophages for the inflammatory response in acute liver injury. Front Physiol.

[41] Elaraj DM, Weinreich DM, Varghese S, Puhlmann M, Hewitt SM, Carroll NM, Feldman ED, Turner EM, Alexander HR (2006). The role of interleukin 1 in growth and metastasis of human cancer xenografts. Clin Cancer Res.

[42] Lavi G, Voronov E, Dinarello CA, Apte RN, Cohen S (2007). Sustained delivery of IL-1Ra from biodegradable microspheres reduces the number of murine B16 melanoma lung metastases. J Control Release.

[43] Arend WP (2002). The balance between IL-1 and IL-1Ra in disease. Cytokine & growth factor Rev.

[44] Masanori KU, Toshiko KO, Maune H, Fukuda T, Azuma J (2007). Pharmacokinetics of aripiprazole, a new antipsychotic, following oral dosing in healthy adult Japanese volunteers: influence of CYP2D6 polymorphism. Drug Metab and Pharmacokinet.

[45] Azuma J, Hasunuma T, Kubo M, Miyatake M, Koue T, Higashi K, Fujiwara T, Kitahara S, Katano T, Hara S (2012). The relationship between clinical pharmacokinetics of aripiprazole and CYP2D6 genetic polymorphism: effects of CYP enzyme inhibition by coadministration of paroxetine or fluvoxamine. Eur J Clin Pharmacol.

[46] Mahgoub AA (2002). Grapefruit juice potentiates the anti-inflammatory effects of diclofenac on the carrageenan-induced rat’s paw oedema. Pharmacol Res.

[47] Hatia S, Septembre-Malaterre A, Le Sage F, Badiou-Beneteau A, Baret P, Payet B, Lefebvre d’hellencourt C, Gonthier M (2014). Evaluation of antioxidant properties of major dietary polyphenols and their protective effect on 3T3-L1 preadipocytes and red blood cells exposed to oxidative stress. Free Radic Res.

[48] Wang S, Moustaid-Moussa N, Chen L, Mo H, Shastri A, Su R, Bapat P, Kwun I, Shen C-L (2014). Novel insights of dietary polyphenols and obesity. J Nutr Biochem.

